# Conceptual causal framework to assess the effect of SARS-CoV-2 variants on COVID-19 disease severity among hospitalized patients

**DOI:** 10.1186/s13690-021-00709-x

**Published:** 2021-10-25

**Authors:** Nina Van Goethem, Ben Serrien, Mathil Vandromme, Chloé Wyndham-Thomas, Lucy Catteau, Ruben Brondeel, Sofieke Klamer, Marjan Meurisse, Lize Cuypers, Emmanuel André, Koen Blot, Herman Van Oyen

**Affiliations:** 1 Scientific Directorate of Epidemiology and public health, Sciensano, J. Wytsmanstraat 14, 1050 Brussels, Belgium; 2 Department of Epidemiology and Biostatistics, Institut de recherche expérimentale et clinique, Faculty of Public Health, Université catholique de Louvain, Clos Chapelle-aux-champs 30, 1200 Woluwe-Saint-Lambert, Belgium; 3 Department of Laboratory Medicine, National Reference Centre for Respiratory Pathogens, University Hospitals Leuven, Herestraat 49, BE-3000 Leuven, Belgium; 4 KU Leuven, Department of Microbiology, Immunology and Transplantation, Rega Institute for Medical Research, Laboratory Clinical Bacteriology and Mycology, Herestraat 49, box 1040, BE-3000 Leuven, Belgium

**Keywords:** COVID-19, SARS-CoV-2 variants, Hospitals, Causality

## Abstract

**Background:**

SARS-CoV-2 strains evolve continuously and accumulate mutations in their genomes over the course of the pandemic. The severity of a SARS-CoV-2 infection could partly depend on these viral genetic characteristics. Here, we present a general conceptual framework that allows to study the effect of SARS-CoV-2 variants on COVID-19 disease severity among hospitalized patients.

**Methods:**

A causal model is defined and visualized using a Directed Acyclic Graph (DAG), in which assumptions on the relationship between (confounding) variables are made explicit. Various DAGs are presented to explore specific study design options and the risk for selection bias. Next, the data infrastructure specific to the COVID-19 surveillance in Belgium is described, along with its strengths and weaknesses for the study of clinical impact of variants.

**Discussion:**

A well-established framework that provides a complete view on COVID-19 disease severity among hospitalized patients by combining information from different sources on host factors, viral factors, and healthcare-related factors, will enable to assess the clinical impact of emerging SARS-CoV-2 variants and answer questions that will be raised in the future. The framework shows the complexity related to causal research, the corresponding data requirements, and it underlines important limitations, such as unmeasured confounders or selection bias, inherent to repurposing existing routine COVID-19 data registries.

**Trial registration:**

Each individual research project within the current conceptual framework will be prospectively registered in Open Science Framework (OSF identifier: 10.17605/OSF.IO/UEF29). OSF project created on 18 May 2021.

## Background

The pathogenesis of SARS-CoV-2 infection ranges from mild symptoms to severe respiratory failure [1]. Host factors play an important role in COVID-19 disease severity. Many studies have already identified older age and certain comorbidities, such as chronic immunocompromised conditions, chronic kidney disease, cardiovascular disease, diabetes mellitus, and obesity, as risk factors for hospitalization and mortality [2–5]. In addition, genetic association studies have identified several host genetic risk factors to become severely ill when infected by SARS-CoV-2 [6], including genetic variants in genes related to the immune system, such as the Human Leukocyte Antigens (HLA) gene complex [7] and cytokine genes, or in genes encoding human receptors of SARS-CoV-2 [8], such as ACE2 [9] and TMPRSS2 [10–12]. COVID-19 vaccines have proven to be highly effective against laboratory-confirmed SARS-CoV-2 infections and COVID-19 hospitalizations, severe disease, and deaths [13–19]. Whilst the vaccine effectiveness is shown at the population level, individual responses to vaccines will differ as a result of host factors and external factors [20, 21]. Next to vaccination and other factors related to the host, severity of outcome can be influenced by aspects related to the healthcare organization and patient management [5, 22, 23].

Finally, severity of a SARS-CoV-2 infection could depend on the viral genetic characteristics. For other viruses such as influenza, it is well documented that viral genetic variation plays an important role in pathogenicity [24–28]. SARS-CoV-2, as other RNA viruses, evolves continuously via point mutations, deletions, insertions and possibly re-assortments resulting in an expanding phylogenetic diversity. This genetic diversity can lead to the emergence of new variants with specific characteristics. Most emerging mutations will not provide a selective advantage to the virus, however some circulating variants may have increased viral fitness and are consequently labeled as ‘Variant of Concern (VOC)’ [29]. When the emerging variant possesses a selective advantage, it may dominate other circulating variants as time goes by [30]. Whole-genome sequencing (WGS) of the SARS-CoV-2 genome has been extensively applied during the COVID-19 pandemic [31, 32] and rapid public sharing of sequences allowed researchers to look at associations between SARS-CoV-2 genomic variants and disease severity [33–41]. However, these analyses have often remained inconclusive due to small sample sizes, sampling biases, limited availability of detailed patient data and the inability to appropriately adjust for potential confounding factors. Given these encountered limitations that are inherent to observational studies based on real-life data, making causal claims is challenging and requires transparency of assumptions and corrections through study design and statistical analyses. All factors listed above should be studied within one framework as they all influence disease outcomes among COVID-19 patients.

In this manuscript, we present a conceptual framework that allows to study the effect of SARS-CoV-2 variants on COVID-19 disease severity among hospitalized patients. First, we construct a causal model in a general hospital setting and identify important confounders, assess potential selection bias and state the underlying assumptions. Next, the causal model is translated into data needs and we describe the structure of the data collection that is specific to the surveillance of COVID-19 in Belgium, as well as the corresponding architecture linking the different data sources which allows to combine information on the viral genome, host characteristics, vaccination status, clinical outcomes, and other factors such as healthcare organization.

## Methods

### Definition of exposure and outcome

SARS-CoV-2 genetic variation can be defined on different levels, being SARS-CoV-2 lineages, clades, protein-level mutations, or single nucleotide polymorphisms (SNPs). WGS, or at least Sanger sequencing of selected parts of the viral genome, should be performed to confirm infection with a specific variant. Alternative methods, such as diagnostic PCR-based assays, can also be used as an indicator or screening method for particular VOCs. However, results from the latter should not be over-interpreted as they only identify specific mutations, and fail in definitive confirmation of a VOC or non-VOC variant [42]. The genetic variants of interest will change over the course of the epidemic; yet, the study protocol can and should stipulate *a priori* hypotheses to avoid fishing expeditions. For simplicity, we will denote the exposure of interest as ‘variant’. Similarly, the outcome ‘severity’ is a broad denominator that should be clearly defined before starting any analysis. Examples of severity indicators among hospitalized patients are admission to an intensive care unit, the use of invasive ventilation or extracorporeal life support (ECLS), acute respiratory distress syndrome (ARDS), length of stay in the hospital, or mortality.

### Description of the causal model

**Fig. 1:**
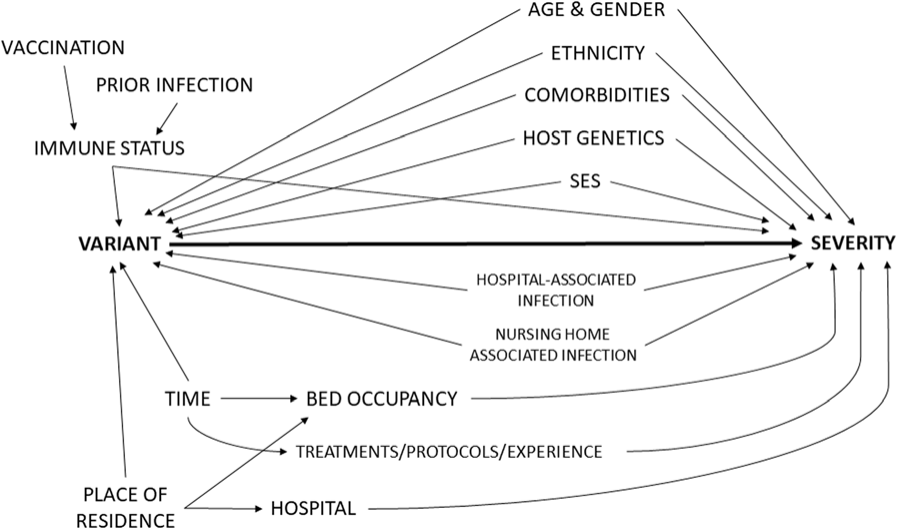
Conceptual framework to assess the causal relationship between SARS-CoV-2 variants and COVID-19 disease severity among hospitalized patients presented in a Directed Acyclic Graph (DAG). VARIANT = infection with a particular SARS-CoV-2 variant. SEVERITY = developing severe complications following SARS-CoV-2 infection. SES = socio-economic status

We use a Directed Acyclic Graph (DAG) to explicitly state the underlying assumptions that are made to estimate the causal effect of infection with a SARS-CoV-2 variant on COVID-19 disease severity among hospitalized patients [43]. The DAG represents both observed and unobserved random variables. Arrows in the DAG denote direct causal effect, while the absence of an arrow between two variables represents the assumption of no causal effect. More details on causal inference and basic DAG concepts can be found elsewhere [43–45]. The DAG in Fig. 1 comprises our qualitative causal assumptions using background knowledge based on the literature and expert opinion.

The probability of infection with a SARS-CoV-2 variant depends among others on the age, gender, ethnicity, host genetics, immune status, comorbidities, and socio-economic status (SES) of the patient [38, 46, 47]. These factors may also influence the severity of COVID-19 disease and can therefore be considered as common causes of the exposure-outcome relationship. Adjusting for these confounders is necessary in order to obtain an unbiased estimate of the effect of variant on severity. For example, if we assume that host genetics influence both the chance of being infected with a particular SARS-CoV-2 variant and the chance to develop severe complications, omission of this variable could potentially invalidate the conclusions. In the reality of an observational design, we will be faced with unmeasured factors and we will not be able to adjust for all variables that we consider as potential confounders.

Further, we assume that the increased circulation of a SARS-CoV-2 variant within the general population might be associated with a peak in cases [47] and a subsequent surge in hospital admissions due to increased transmissibility and/or severity, or due to coincidence [40]. Consequently, the circulation of a SARS-CoV-2 variant and a high occupancy rate of hospital beds reserved for COVID-19 patients are possibly linked to the stage of the epidemic (time) within a given geographical region (place of residence) [48]. We learned from previous analyses that overflow of recognized intensive care unit (ICU) beds negatively impacts the outcome of the patient [5]. In order to estimate the causal effect of a SARS-CoV-2 variant on severity, we therefore need to adjust the analysis for bed occupancy to avoid a spurious association between variant and severity that flows from variant via time or place of residence to bed occupancy and finally to severity.

In addition, the circulation of a variant can coincide in time with changes in treatment guidelines, protocols and experience. A spurious association between variant and severity can be prevented by adjusting for time, which could for example be defined by “wave”. This will block the backdoor pathway from variant to treatments/protocols/experience through time to severity. Previous analyses also showed that patient outcomes significantly vary between hospitals [5, 49]. Patients are more likely to be admitted to a hospital in close proximity to their place of residence. The increased circulation of a variant could coincide in place with the location of a hospital. Therefore, a spurious association between variant and severity should be prevented by adjusting for place or adjusting for the hospital where the patient is treated.

Similarly as for time and place of residence, the circulation of SARS-CoV-2 variants may differ between settings that are relatively disconnected from each other. The risk to be infected with a particular variant within a healthcare setting, such as a hospital or nursing home, is potentially different from the risk within the community. Healthcare-associated COVID-19 infections have been shown to be associated with higher risk of clinical deterioration (requirement of ventilatory support, critical care or death), likely because hospitalized patients represent a frailer population with decompensated comorbidities compared to the community [23]. Consequently, the analysis should be adjusted for infections contracted within the hospital or within a nursing home.

### Blocking non-causal associations

**Fig. 2:**
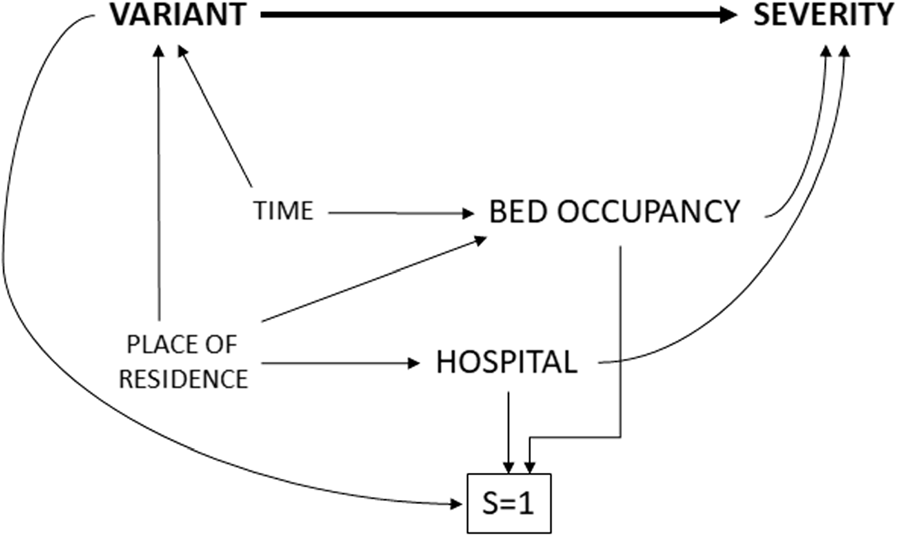
Blocking non-causal associations between SARS-CoV-2 variants and COVID-19 disease severity among hospitalized patients by the use of a matched cohort design presented in a Directed Acyclic Graph (DAG). S = selection into the study

After identifying the sufficient set of variables from the DAG, we can remove the bias resulting from non-directed open paths by covariate adjustment in a regression analysis and subsequently identify the targeted causal path between variant and severity. Alternatively, we can consider balanced matching to force the distribution of the matching factors to be identical across the exposed and unexposed groups. Figure 2 represents a DAG of a possible matched cohort design within the conceptual framework where the exposed and non-exposed are matched based on similar levels of care, i.e. hospital and bed occupancy. The variable S indicates whether an individual in the source population is selected for the matched study. Most cohort matching starts with exposed individuals, i.e. those infected with an emerging SARS-CoV-2 variants, and subsequent selection of unexposed individuals with the same values for the matching values. As a results, the exposed are more likely to be selected into the matched sub-cohort as represented by the arrow from variant to S. Unexposed individuals that are not matched to the exposed are usually discarded from the analysis (i.e. analysis is restricted to the matched subset) and hence the study sample has proportionally more patients exposed to the emerging variant than in the source population. The arrow from the bed occupancy and hospital to S indicate that among the unexposed, individuals will be selected based on their values for bed occupancy and hospital. Matching (i.e. conditioning on S represented by a square box) induces an association via the path occupancy/hospital to S to variant that is of equal magnitude, but opposite direction, to the association via the path occupancy/hospital to variant (through time/place), ensuring that bed occupancy and hospital are both independent from variant in the matched sub-cohort. Because of the matching, the joint distribution of the matched data does not follow the causal structure for the source population as presented in the DAG, in the sense that the paths between variant and hospital and between variant and occupancy through time and place are no longer present in the matched sub-population (i.e. unfaithfulness [50]).

### Identification of selection bias

**Fig. 3:**
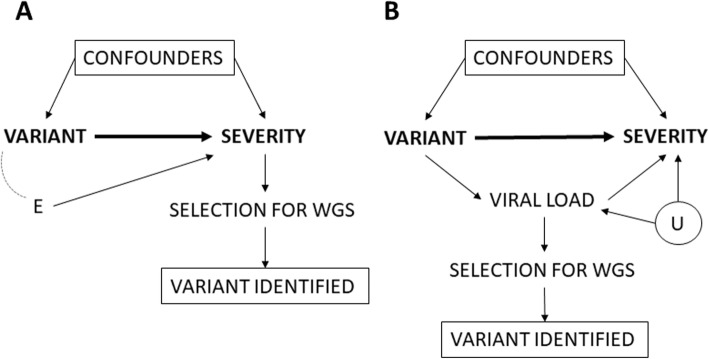
Conceptual framework to assess the causal relationship between SARS-CoV-2 variants and COVID-19 disease severity among hospitalized patients presented in a Directed Acyclic Graph (DAG) in the scenario of selection bias. CONFOUNDERS = all other confounders as listed in the DAG in Fig. 1. E = error term. U = unmeasured confounders

Even in the scenario in which it would be possible to adjust the analysis for all important confounders as depicted in the DAG (Fig. 1), there can still arise a spurious association between variant and severity due to selection bias (Fig. 3). WGS is not performed on the samples of all COVID-19 patients, but on a selection of them. Subsequently, only patients with known exposure, i.e. information on the SARS-CoV-2 variant that they are infected with, will be included in the study. Figure 3A represents the situation in which the selection of samples for WGS depends on the severity of the patient, i.e. conditioning on the outcome, which is potentially problematic and is often denoted as sample truncation bias [51]. Severity is affected by the variant, as well as by other factors which are subsumed in an error term E. Indeed, severity is a collider variable (i.e. a common effect) on the path between variant and the error term E, and conditioning on severity induces a spurious association between variant and E in the selected sample, even if they are independent in the general population. As such, restricting the sample to severe patients results in a newly induced non-causal path that flows from variant to E to severity. Figure 3B represents the situation when the total causal effect cannot be estimated in the case of conditioning (i.e. selection) on an intermediate variable. Due to analytic quality reasons it is recommended to only select samples for sequencing with a sufficient high viral load (≥10^3^–10^4^ RNA copies/mL) [52]. Conditioning on samples with a high viral load blocks the causal path of variant on severity that is mediated by the viral load [53] leading to overcontrol bias [51]. In addition, conditioning on viral load may induce a spurious association between variant and severity when there exists an unmeasured confounder (U) of the relationship between viral load (i.e. the mediator) and severity (i.e. the outcome).

### Translation into data requirements

**Fig. 4:**
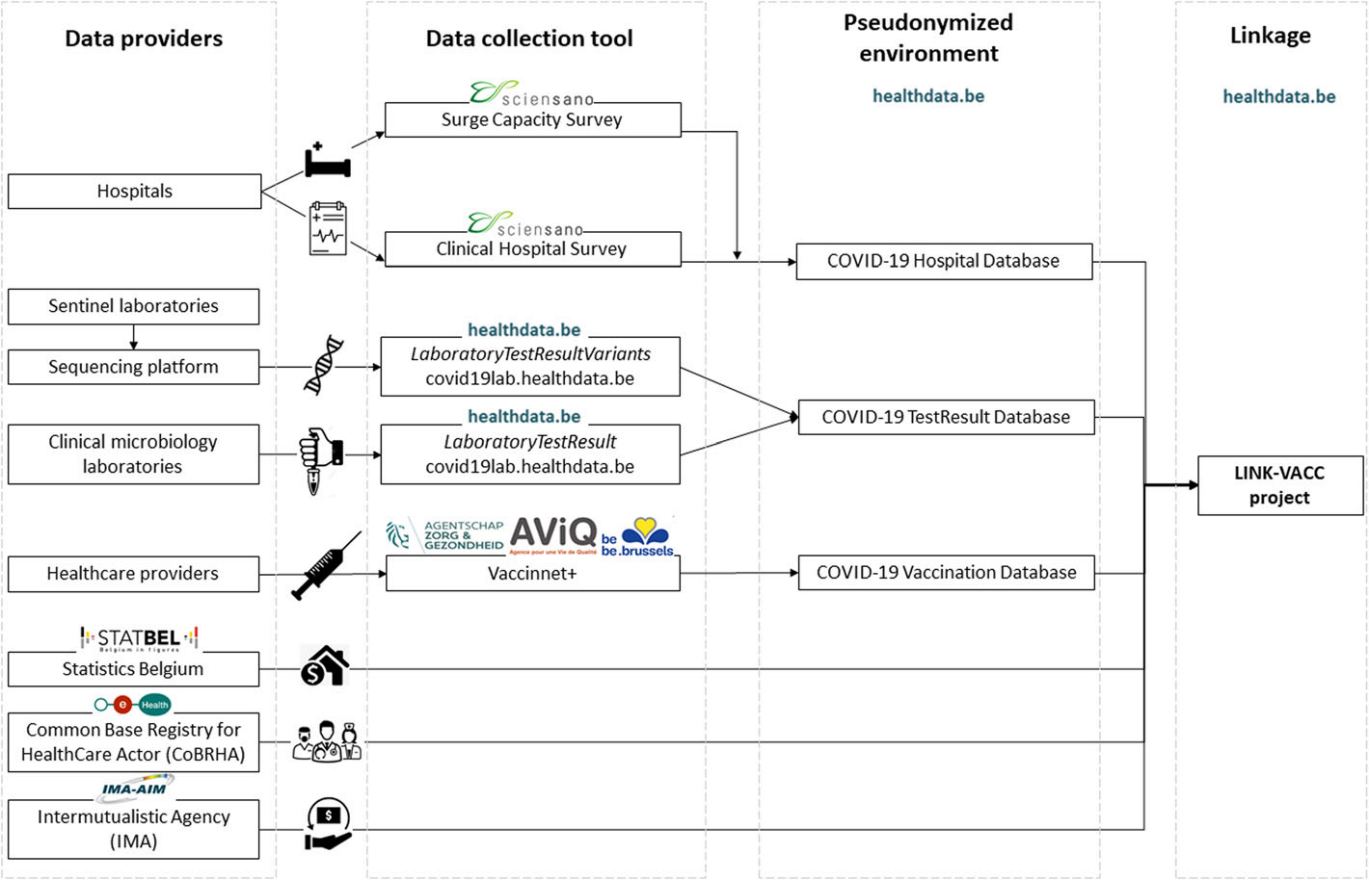
Data linkage of existing COVID-19 surveillance registries within the context of the LINK-VACC project, Belgium

The causal model is translated into data requirements in order to meet the assumptions as depicted in the DAG (Fig. 1). In the context of the COVID-19 pandemic, Sciensano, the Belgian institute for health, has been mandated to describe the evolution of the epidemic and assess its consequences on the health of the Belgian population. Sciensano collects data on laboratory-confirmed COVID-19 cases, testing, hospitalized COVID-19 patients, COVID-19 deaths and COVID-19 vaccinations. These surveillance systems have in common that they channel data flows from one or more sources that reach Sciensano either directly or via an intermediate step. Amongst others, Sciensano has launched the LINK-VACC project, which aims at linking selected variables from existing registries for COVID-19 vaccine surveillance. The IT architecture of the LINK-VACC platform, hosted by the healthdata.be service of Sciensano, is used to organize data transfers to store and to link the different data sources based on the national registry number within a pseudonymized environment. The present framework takes place within the architecture of the LINK-VACC project which allows to meet the assumptions of the causal model by combining selected variables from multiple data sources merged on the individual patient level (Fig. 4). The protocol of the LINK-VACC project was approved by the medical ethics committee University Hospital Brussels – Vrije Universiteit Brussel (VUB) on 03/02/2021 (reference number 2020/523) and obtained authorization from the Information Security Committee (ISC) Social Security and Health (reference number IVC/KSZG/21/034).

I. Data on hospitalized COVID-19 patients

Data on hospitalized COVID-19 patients is collected through two complementary surveillance systems [54]. The Surge Capacity Survey (SCS) exhaustively collects data on the number of COVID-19 patients at an aggregated level per hospital and enables calculation of the daily occupancy rate of beds reserved for COVID-19 patients per hospital accreditation number. The Clinical Hospital Survey (CHS) collects individual data of patients hospitalized with confirmed COVID-19 through an admission, discharge and detailed ICU form. The CHS is not exhaustive as participation is voluntary and covers approximately 65% of all hospitalized COVID-19 patients in Belgium. However, the CHS is considered to be representative as it covers all provinces in Belgium and includes public and private, academic and non-academic hospitals.

II. Data on COVID-19 test results

The COVID-19 TestResult Database, hosted by the Sciensano service Healthdata.be [55], collects the RT-PCR and antigen test results from clinical microbiology laboratories (CML) and physicians since the 5th of May 2020, including retrospective data collection since February 2020 [56]. The test results are accompanied by the date of sampling, the date of test result, the test result, the type of diagnostic test, a sample identification number, the laboratory identification number and patient demographic variables. Daily reporting of all test results of RT-PCR and antigenic diagnostic tests is mandatory for reimbursement.

III. Data on COVID-19 sequencing results

The National Reference Center (NRC) of respiratory diseases has put in place genomic surveillance at the national level since the first introduction of the virus, together with other university centers. The genomic surveillance of SARS-CoV-2 has scaled-up from December 2020 onwards resulting in a federal sequencing consortium currently including a total of 17 laboratories. Given the emergence of VOCs in December 2020, the samples selected for sequencing at that time resulted mainly from active surveillance focusing on returning travelers, atypical PCR results and large outbreaks. Subsequently, baseline surveillance was set up in January 2021 to obtain a representative sample of the positive cases. This is obtained through collaboration with a sentinel network of laboratories that send a proportion of their positive samples to one of the laboratories that are part of the sequencing consortium to ensure an optimal geographical coverage and a diversity of clinical severity patterns. The aim is to cover approximately 10% of all positive cases in Belgium. The baseline surveillance is complemented by targeted active surveillance that focuses on the systematic screening of patients experiencing re-infection, vaccine breakthrough cases, immunocompromised patients, and a selection of samples linked to outbreaks, returning travelers from red zones and atypical PCR results [52]. In addition, hospitals and laboratories are allowed to perform additional sequencing outside reimbursement indications on own initiative: some hospitals took the initiative to systematically sequence all available positive samples from patients admitted to ICU, while other hospitals aim to sequence exhaustively all available SARS-CoV-2 positive samples from their hospitalized patients.

The reporting of variant information from sequencing laboratories to Sciensano through h-Healthdata.be has been put in place since March 2021. For this purpose, an additional message (“LaboratoryTestResultVariants”) is to be sent to the Central COVID-19 Database [57]. After sequencing, the SARS-CoV-2 variants are assigned a Pangolin lineage, the nomenclature system for SARS-CoV-2 that has been put in place by Rambaut et al [58], and are subsequently designated as confirmed SARS-CoV-2 variants. Besides information on confirmed variants obtained through sequencing, the laboratories also report test results from diagnostic SNP assays to detect samples compatible with known VOCs. The collected information consists of the indication of the reason for which the sample was selected for sequencing (baseline or active surveillance), the mutations tested in case of an RT-PCR SNP assay, the mutations and deletions detected in the S gene, and in case of sequencing: the Pangolin lineage and the GISAID accession number.

IV. Data on COVID-19 vaccines

As defined by the Belgian law, all COVID-19 vaccines administered in Belgium are recorded in Vaccinnet+, the national vaccine registry. Vaccination data are subsequently sent to Healthdata.be at Sciensano. Researchers at Sciensano have access to a pseudonymized version of these data including demographic data of the vaccinated person (age, gender and place of residence) and information on the administered vaccine (brand, lot number, administration date and registration date).

V. Other data sources

Within the LINK-VACC environment, data from existing registries outside Sciensano will also be linked on the individual patient level using the national registry number. Statbel, the Belgian statistical office, collects and shares data on the Belgian economy, society and territory. Individually-linked data will allow to retrieve socio-economic information (e.g. civil status, employment status and income decile), identify residents of collectivities, and obtain information on long-term survival and cause of death. The Intermutualistic Agency (IMA) collects data on reimbursed care and medications of citizens insured in Belgium and will enable to provide information on comorbidities and pregnancy status, but also give insights on long-term healthcare and medication consumption (e.g. after COVID-19 hospitalization). The Common Base Registry for HealthCare Actor (CoBRHA) enables to identify Belgian healthcare workers by profession type based on their license to practice.

### Study population

**Fig. 5:**
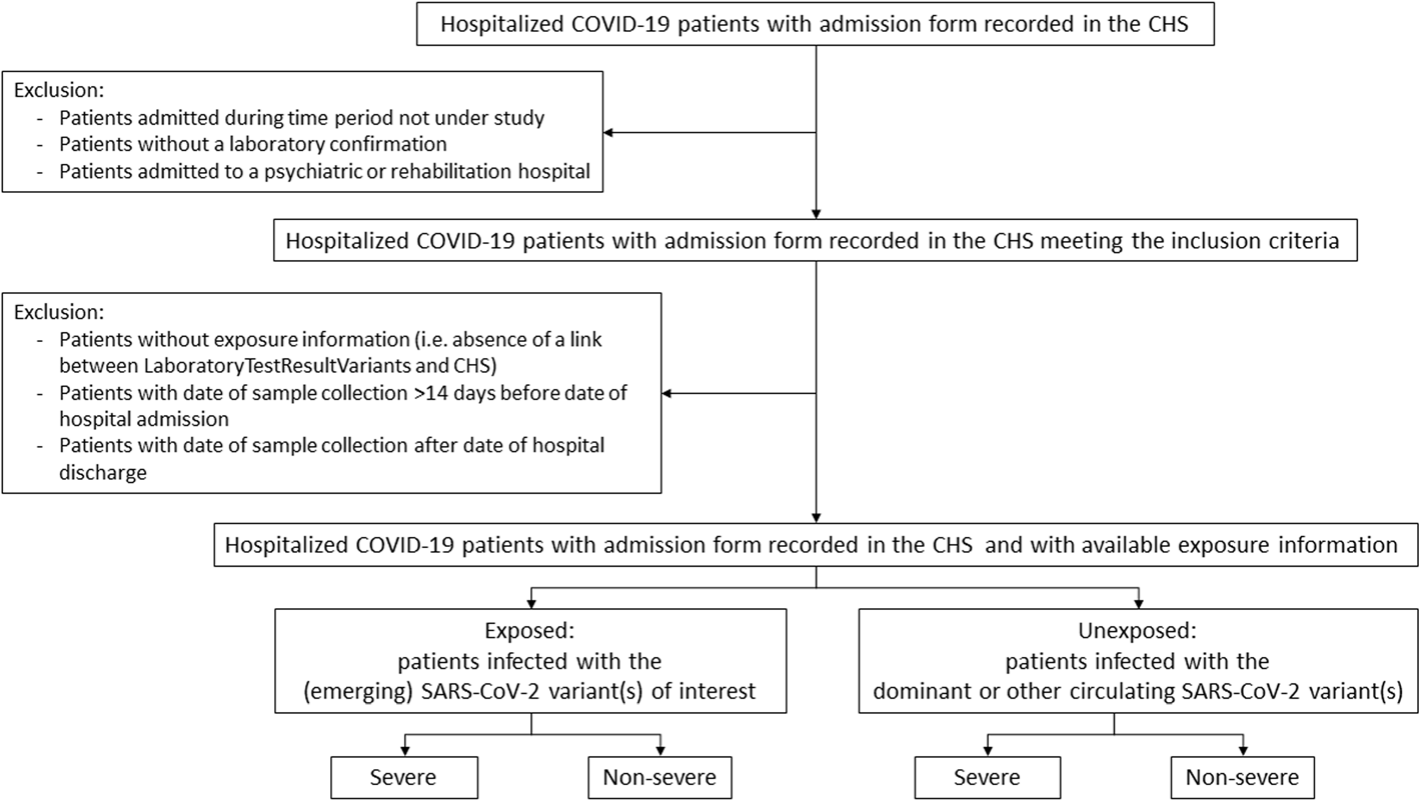
Flow chart of the study population selection to study the effect of SARS-CoV-2 variants on COVID-19 disease severity among hospitalized patients. CHS = Clinical Hospital Survey

The study population consists of hospitalized COVID-19 patients with an available admission form registered in the CHS and with information available on the SARS-CoV-2 variant (exposure) of their infection as obtained through the linkage of the CHS database with the COVID-19 TestResult Database (Fig. 5). The choice to study severity among hospitalized patients is supported by the detailed information on patient characteristics and the well-defined severity indicators available in the CHS data collection. Patient information related to positive cases is limited to demographics (age, gender, and residence) and is not suitable to study severity of disease. In addition, defining severity based on hospitalization itself is hampered by the fact that information on hospital admission is derived from the non-exhaustive registration of hospitalized COVID-19 patients in the CHS. As a consequence, absence of registration of a patient in the CHS does not imply that this patient was not admitted to the hospital.

### Study design

The study is an observational multi-center cohort study where COVID-19 hospitalized patients are followed-up from hospital admission until death or hospital discharge and for whom information is obtained by merging different national surveillance systems based on the national registry number. Within the described framework, several research questions, study designs and analyses are possible and should be defined on a case-by-case basis depending on the requirements at a specific time of the epidemic.

### Data analysis plan

The definition of the outcome will define the statistical model being used. A logistic regression or log-binomial model may be used for binary outcomes and a survival analysis for time-to-event data. Selection of main effects in the model will be based on the minimal sufficient adjustment set of covariates as identified from the DAG. The model with all main effects will be compared to models including interaction terms between the exposure and covariates or including non-linear terms for continuous covariates. Regression standardization may be used to estimate the causal effects of interest. Alternative to standardization, we may use inverse-probability weighting and doubly-robust methods [43]. The modeling approach should account for the clustering effects within hospitals, for example by matching on hospital or by using generalized estimating equation (GEE) models. Sensitivity analyses can be used to assess robustness to unmeasured confounding, selection bias or measurement error [59]. If a matching procedure is used, the matching factors do not require adjustment in the model as they are already accounted for by the design. Multiple imputation will be used for missing data on the outcome or potential confounders. As the emergence of VOCs in Belgium occurred from December 2020 onwards, patients admitted before 1/12/2020 are classified as being exposed to “previously circulating SARS-CoV-2 variants”. Vaccination rollout began in January 2021 and initially focused on nursing home residents and healthcare workers. From March 2021 onwards, people were vaccinated according to their age group. Therefore, patients admitted in the year 2020 are classified as non-vaccinated. A detailed data analysis plan will be anticipated for each specific research question addressing a particular exposure-outcome relationship. Each individual research project will be registered in Open Science Framework (https://osf.io/zg3dj)

## Discussion

This manuscript presents a conceptual framework to study the effect of SARS-CoV-2 variants on COVID-19 disease severity among hospitalized patients. A DAG was used to explicitly state the underlying assumptions of the causal model. Next, the DAG was translated into the data requirements, i.e. the necessary information on important confounders, that would allow to obtain an unbiased estimate of the causal effect of SARS-CoV-2 variants on severity of COVID-19 disease. The current framework takes advantage of the LINK-VACC architecture to combine information from existing surveillance registries on the viral genome, host characteristics, vaccination status, clinical outcomes, and other factors such as healthcare organization. As such, it allows to perform a cohort study among hospitalized COVID-19 patients, for whom information on the exposure, outcome and confounders is obtained by merging existing national surveillance systems based on the national registry number, to study the clinical impact of SARS-CoV-2 variants. Within the described framework, several research questions, study designs and analyses are possible and should be defined on a case-by-case basis depending on the requirements at a specific time of the epidemic.

The conceptual framework underlines important limitations of the current data architecture. First, not all confounders are correctly measured or even measured at all and the resulting bias should be quantified to assess robustness of the conclusions [59]. Host genetic variants may partially account for the residual variability in disease severity between patients after adjusting for other risk factors such as age and comorbidities [12, 60]. Further, it is not unlikely that host genetic variants influence the probability to be infected with a particular SARS-CoV-2 variant given the importance of host-pathogen interactions [61]. Collecting information on the ethnicity of the patient may partially account for this bias [62–64], but residual confounding is likely to exist. Genetic profiling to discover patients’ increased susceptibility to life-threatening conditions caused by an infectious disease such as COVID-19 has been put forward as one of the priorities in the 1+ Million Genomes Roadmap. However, integration of omics data in public health remains limited given the challenges related to the generation, analysis and interpretation of high-dimensional data [65] and potential privacy and security concerns. Next to the absence of information on confounders that should be adjusted for, the analysis can also suffer from inaccurate measurements or partial information on certain confounders. For example, the occupancy rate of ICU beds is currently calculated based on the number of COVID-19 patients in the hospital and the number of recognized ICU beds available in the hospital reserved for COVID-19 patients. However, the latter also depends on the load of non-COVID-19 patients which may vary between hospitals and the different waves according to the directives in force. Information on the number of non-COVID-19 patients and their impact on total hospital or ICU capacity is less well documented. In addition, quality of care not only depends on occupancy rate, but also on the staff-to-patient ratio and the professional experience of healthcare workers.

Second, a potential threat for the current conceptual framework is the selection bias that arises when there is an increased focus on selecting samples for WGS from patients that have developed severe complications. As a consequence, the selection bias will have a strong effect on the representativeness as the study population will not represent the full spectrum of hospitalized COVID-19 patients. In addition, selection bias can induce collider bias (which occurs after conditioning on a common effect) which can lead to substantially biased estimates of associations [66]. This underlines the importance of obtaining a representative and unbiased SARS-CoV-2 sequence collection. Both the European Centre for Disease Prevention and Control (ECDC) [67] and the World Health Organization (WHO) [68] have provided guidance on representative sampling and sequencing of SARS-CoV-2 cases from routine surveillance. In Belgium, genomic surveillance consists of a passive component that aims to obtain a representative set of sequences based on a network of sentinel laboratories, and an active component, including targeting sequencing of vaccine breakthrough cases and a selection of samples from outbreaks and returning travelers. In addition, there has been an increased focus on sequencing samples from COVID-19 patients admitted to an intensive care unit. The data collection tool contains a variable “indication for WGS” which allows to differentiate between samples obtained from the active and passive surveillance arm and can hence be used to eliminate the selection bias. Even if there is no selection bias among the hospitalized patients, the current framework only allows to estimate the risk associated with a particular SARS-CoV-2 variant on severe clinical evolution once hospitalized. Estimating the effect of SARS-CoV-2 variants on disease severity among the general population of COVID-19 patients requires linkage with an exhaustive data source of hospital admission data based on the national registry number.

Here, we have established a framework to study the exposure-outcome relationship by merging data from routine national surveillance systems. Observational studies that aim to estimate a causal effect are often faced with an imbalance of baseline characteristics of patients between groups. Adjustment for confounding variables can be accomplished by including them in a regression model or by conducting a matched cohort study [69]. Matching aims to balance the groups with respect to factors which may influence the outcome. However, if the matching ratio is low, the matching design may suffer from loss of efficiency as the analysis is restricted to a subset of patients. Also, if interaction effects between the exposure and covariates are of interest and the objective of the study, these covariates cannot be used in the matching criterion.

The study population consists of a cohort of hospitalized COVID-19 patients that are registered in the CHS and with available SARS-CoV-2 variant information. The sample size is therefore limited by the presence of the patient in the two independent data sources. The advantage of this strategy is that re-using existing data sources avoids investing resources in setting up additional data collection systems. However, the ability to provide an answer to our research questions heavily depends on the features of the existing data sources. Given that the data collection system was not specifically designed for the requirements of the current analysis, it may result in potential threats to the causal model of interest, such as unmeasured confounding and selection bias. However, the anticipation of future research questions is a challenging task as research interests typically change over the course of a pandemic. This underlines the importance of the establishment of a versatile framework that allows researchers to efficiently combine data from different sources and which is flexible to be adjusted for different purposes. An alternative approach for the selection of samples for sequencing that would circumvent the selection bias and the relatively small sample size related to linkage of existing data sources is to conduct a *nested* case-control study within the cohort of hospitalized patients registered in the CHS. Several methods to sample controls can be applied. For example, one can randomly select a control each time a case is diagnosed. Another possibility is to sample the control group at the beginning of the follow-up period resulting in a sample that is representative of the full cohort from which all future cases will develop. This type of *nested* case-control study is usually referred to as a case-cohort study. If the controls are indeed a representative sample of the study base, the exposure odds ratio is a valid estimate of the incidence rate ratio one would obtain from a cohort study. In addition, a case-cohort design has the advantage that a single control group can be applied for multiple outcomes. A careful selection of cases and controls and the subsequent sequencing of the samples of these patients allows an efficient use of resources. Indeed, a nested case-control study design is particularly appealing when the assessment of the exposure is expensive. However, a retrospective selection for WGS requires the preservation of samples, i.e. by bio-banking or mid-term storage of the samples of all hospitalized patients.

As a final remark, statistical analyses under the proposed causal model require considerable sample sizes which can take a while to accumulate while a new variant emerges. After the arrival of a new variant, it takes several weeks before it becomes dominant and/or before it reaches the more vulnerable population that has a high risk of hospitalization. In addition, we should take into account the length of stay and reporting delay before the hospital discharge data is recorded in the surveillance system. As a consequence, rapid assessments of the clinical impact of new emerging SARS-CoV-2 variants among hospitalized patients is challenging. The uncertainty related to small sample sizes in the early phases of a new emerging variant should be acknowledged by researchers and policy makers in their communication to the public.

## Conclusion

A well-established framework that brings together information from different domains and thereby provides a complete view on the factors that influence COVID-19 disease severity will enable to assess the impact of emerging SARS-CoV-2 variants and answer questions that will be raised in the future. This conceptual framework is important as a theoretical foundation for the development of routine clinical epidemiological research and may serve as a basis for future pandemics. An evaluation and update of the framework should be conducted regularly in terms of emerging new viral, social or clinical trends or when a new data architecture allows for improved analyses.

## Data Availability

The data obtained through the surveillance systems are available from the corresponding author on reasonable request according to Sciensano scientific policy and after approval by the Belgian data protection authority.

## Abbreviations

ACE2: Angiotensin converting enzyme 2
ARDS: Acute respiratory distress syndrome
CHS: Clinical hospital survey
CML: cliNical microbiology laboratories
CoBRHA: Common Base Registry for HealthCare Actor
COVID-19: Coronavirus disease 2019
DAG: Directed acyclic graph
ECDC: European Centre for Disease Prevention and Control
ECLS: Extracorporeal life support
GDPR: General Data Protection Regulation
GEE: Generalized estimating equation
GISAID: Global initiative on sharing all influenza data
HLA: Human Leukocyte Antigens
ICU: Intensive Care Unit
IMA-AIM: Intermutualistic Agency
ISC: Information Security Committee
IT: Information Technology
LINK-VACC: Linking of registries for COVID-19 vaccine surveillance
NRC: National Reference Center
RNA: Ribonucleic Acid
RT-PCR: Reverse Transcriptase–Polymerase Chain Reaction
SARS-CoV-2: severe acute respiratory syndrome coronavirus 2
SCS: Surge Capacity Survey
SNP: Single Nucleotide Polymorphism
TMPRSS2: Transmembrane Serine Protease 2
VOC: Variant of Concern
WGS: Whole-Genome Sequencing
WHO: World Health Organization
